# Interactions between Stress and Vestibular Compensation – A Review

**DOI:** 10.3389/fneur.2012.00116

**Published:** 2012-07-27

**Authors:** Yougan Saman, D. E. Bamiou, Michael Gleeson, Mayank B. Dutia

**Affiliations:** ^1^Department of Neuro-otology, National Hospital for Neurology and Neurosurgery, Institute of Neurology, University College LondonLondon, UK; ^2^Ear Institute, University College LondonLondon, UK; ^3^Centre for Integrative Physiology, University of EdinburghEdinburgh, UK

**Keywords:** stress, vestibular compensation, plasticity

## Abstract

Elevated levels of stress and anxiety often accompany vestibular dysfunction, while conversely complaints of dizziness and loss of balance are common in patients with panic and other anxiety disorders. The interactions between stress and vestibular function have been investigated both in animal models and in clinical studies. Evidence from animal studies indicates that vestibular symptoms are effective in activating the stress axis, and that the acute stress response is important in promoting compensatory synaptic and neuronal plasticity in the vestibular system and cerebellum. The role of stress in human vestibular disorders is complex, and definitive evidence is lacking. This article reviews the evidence from animal and clinical studies with a focus on the effects of stress on the central vestibular pathways and their role in the pathogenesis and management of human vestibular disorders.

Stress may influence central vestibular function in health and disease either directly through the actions of glucocorticoids (cortisol and corticosterone) on ion channels and neurotransmission in the brain, or indirectly through the effects of stress-related neuroactive substances (e.g., histamine, neurosteroids) on these structures. In the periphery stress hormones also regulate the function of ion transporters and ionic homeostasis in the inner ear, while in some conditions the anti-inflammatory actions of glucocorticoids may also come into play (Hamid et al., 2009). Stress hormones may thus modulate peripheral vestibular end-organ and cochlear function through similar mechanisms of ionic homeostasis (Canlon et al., 2007; Hamid et al., 2009), and modulate central processing in the vestibular and auditory pathways (Seemungal et al., 2001; Paterson et al., 2004; Straka et al., 2005; Mazurek et al., 2010).

While inner ear lesions often lead to vestibular and auditory deficits in parallel, the extent to which lesions in one system contribute to functional loss and adaptation in the other is at present largely unknown. For example, auditory cues are known to contribute to spatial orientation and balance function in sighted as well as congenitally blind people (Easton et al., 1998; Dozza et al., 2007), and thus auditory loss may be expected to exacerbate vestibular dysfunction. However, although stress influences both the vestibular and auditory systems the mechanisms of plasticity in the central pathways and the interactions between them remain to be systematically investigated.

In relation to vestibular lesions and central vestibular compensation, evidence from animal studies has demonstrated neural pathways linking the vestibular nuclei with the limbic system including the hypothalamus and that stress responses evoked by vestibular symptoms promote synaptic and neuronal plasticity in the vestibular system. However in man conclusive evidence relating to the interactions between stress and vestibular system plasticity is lacking. In patients who remain poorly compensated following vestibular damage, clinical evidence implicates stress both as a causative agent and as a compounding factor that impedes compensation. In parallel the use of steroids in the treatment of acute vestibular symptoms also indicates a role for the acute stress response in facilitating vestibular compensation. Factors that tend to constrain a fuller understanding of the effects of stress in human vestibular disease include sometimes nebulous clinical definitions, difficult to measure endpoints whether clinical or physiological, and variations in the stress response between individuals and within the same individual over time. Nevertheless, the effects of stress on vestibular function and compensation are significant and increasingly recognized as important in the management of vestibular dysfunction in man.

## Stress and Vestibular Compensation in Animal Studies

### Neuroanatomical pathways

The stress response is complex and involves a number of chemical mediators including neuropeptides, steroid, and monoamine hormones (Joels and Baram, 2009). The canonical stress response is mediated by the hypothalamic–pituitary–adrenal (HPA) axis. Corticotrophin releasing hormone (CRH) and arginine vasopressin (AVP) in the paraventricular nucleus (PVN) of the hypothalamus stimulate the release of adrenocorticotropic hormone (ACTH) from the pituitary, which then causes the release of glucocorticoids from the adrenal cortex (Herman et al., 2003).

**Figure 1:**
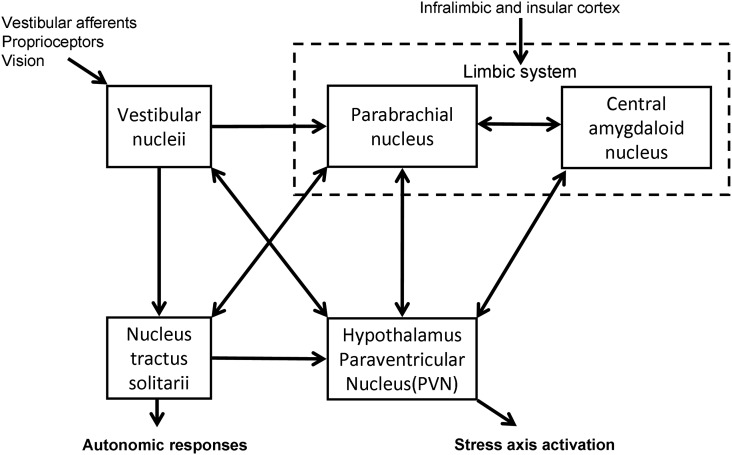
**Diagrammatic representation of the interconnections between the central nervous system structures implicated in stress responsiveness and vestibular dysfunction**.

The vestibular system has been shown to have connections with the HPA axis (Figure 1). Electrical and caloric stimulation of the vestibular pathways results in a response in PVN neurons in the guinea pig (Azzena et al., 1993; Liu et al., 1997) and an increase in plasma AVP levels in the rat (Horii et al., 2001). Retrograde viral tracing in the rat brain has demonstrated the presence of a direct vestibulo-paraventricular projection (Markia et al., 2008) and similarly a paraventricular–vestibular pathway has also been described (Horowitz et al., 2005). Further neural pathways linking the vestibular system and hypothalamus have been suggested in models of the neuroanatomical linkage of balance and anxiety (Balaban and Thayer, 2001) and are supported by recent behavioral experiments in genetically modified mice with either abnormal vestibular systems or altered levels of anxiety-like behavior (Kalueff et al., 2008; Avni et al., 2009). In addition, monoaminergic influences as part of the physiological stress response may be further modulators of these pathways (Balaban and Thayer, 2001; Balaban, 2002, 2004). The central release of histamine for example, is known to stimulate ACTH prolactin and AVP, and to influence vestibular compensation (Bergquist and Dutia, 2006; Bergquist et al., 2006).

### Vestibular dysfunction activates the stress axis

Vestibular symptoms after unilateral vestibular deafferentation (UVD) have been shown to activate the stress axis. Gliddon et al. (2003) measured postoperative cortisol levels in saliva samples from guinea pigs that underwent UVD and animals which underwent a sham operation. Compared to preoperative levels there was a significant increase in salivary cortisol at 10 h post surgery in animals that underwent UVD (Gliddon et al., 2003). In a separate study, Fos expression was demonstrated in the PVN of the hypothalamus after UVD in rats (Cameron and Dutia, 1999).

Tighilet et al. (2009) quantified CRH and AVP expression in hypothalamic neurons in cats 1, 7, 30, and 90 days after unilateral vestibular neurectomy (UVN). An increase was noted at 1, 7, and 30 days post UVN and which returned to normal at 90 days, indicating a long-lasting activation of the HPA stress axis. Behavioral compensation was also measured at the same time points in a separate groups of cats following UVN and a parallel was noted in the recovery from symptoms with the expression of CRH and AVP neurons (Tighilet et al., 2009).

By contrast, Lindsay et al. (2005) and Zhang et al. (2005) assayed serum corticosterone at 10 h and 2 weeks post UVD in three groups of rats either having undergone surgical UVD, sham surgery, or no surgery (anesthetic control), and found no significant difference in serum corticosterone between groups. Lindsay et al. (2005) additionally assessed serum corticosterone at 58 h and found that all groups had a significant decrease in corticosterone, while Zhang et al. (2005) found that the 10-h serum corticosterone was higher in all groups when compared with the 2-week time-point (Lindsay et al., 2005; Zhang et al., 2005).

In contrast to the effects of UVD, following bilateral vestibular deafferentation (BVD) in rats serum corticosterone was not found to be elevated when compared with sham surgery at 3 weeks (Russell et al., 2006) or 6 months (Zheng et al., 2008). Interestingly, the observation that anxiety-like behavior is reduced in animals after BVD (Zheng et al., 2008), suggests that the interactions between the vestibular and limbic systems are chronically altered after vestibular deafferentation.

**Figure 2:**
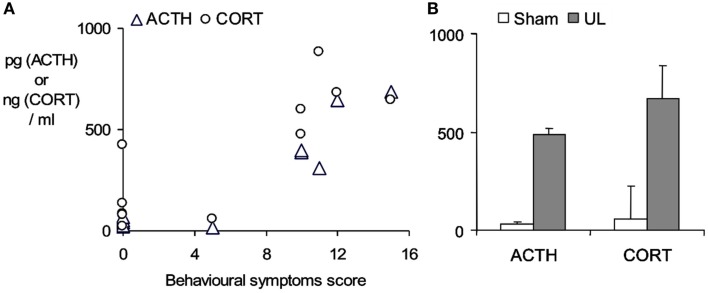
**Stress response evoked by the functional inactivation of the right inner ear by trans-tympanic injection of 3% ropivacaine**. **(A)** ACTH and corticosterone plasma concentrations (pg/ml and ng/ml respectively) measured by radioimmunoassay of trunk blood taken from rats sacrificed 30 min after the onset of the symptoms of unilateral vestibular loss. Symptom severity (spontaneous ocular nystagmus, barrel-rolling, postural asymmetry) was estimated on a behavioral scale as described in detail in Bergquist et al. (2008). Note sham-treated animals (*n* = 5) developed no symptoms and maintained low ACTH and corticosterone levels, while ropivacaine-treated animals (*n* = 6) developed marked behavioral symptoms and showed ACTH and corticosterone levels that were elevated in relation to the severity of the symptoms. **(B)** Mean ± SD of plasma ACTH and corticosterone concentrations in the sham-treated group (open bars) and the ropivacaine-treated group after functional unilateral labyrinthectomy (UL, closed bars). Bergquist, F., Brunton, P., and Dutia, M. B., unpublished data.

An aspect which confounds the characterization of the stress response in studies using a surgical ablation of the labyrinth in UVL or UVN, is the stress imposed by the surgical trauma itself. This may be very largely circumvented by using a trans-tympanic injection of a potent local anesthetic, ropivacaine (3%, under transient general anesthesia), into the middle ear to induce a functional inactivation of the injected ear (Figure 2). Following ropivacaine administration the animals typically develop strong symptoms of unilateral vestibular loss within a few minutes post-injection, which last for several hours. As shown in Figure 2, this also evokes a marked increase in plasma ACTH and corticosterone levels 30 min after the appearance of behavioral symptoms, indicating that the vestibular syndrome is also a stressful condition in itself.

### Stress axis activation facilitates vestibular compensation

In the frog, hypophysectomy delays compensation after UVL while ACTH (4–10) administered post-UVL restores it (Flohr and Luneburg, 1982). In the squirrel monkey ACTH (4–10) given daily post-UVL (Igarashi et al., 1985) and following a two – staged bilateral vestibular labyrinthectomy (Ishii and Igarashi, 1987) was found to improve vestibular compensation. In the guinea pig administration of methylprednisolone (Jerram et al., 1995) and ACTH (4–10; Gilchrist et al., 1990) after UVL were found to improve vestibular compensation while dexamethasone had no effect (Alice et al., 1998). The synthetic ACTH (4–9) fragment (Org 2766) was also shown to improve vestibular compensation following UVL when administered peripherally (Gilchrist et al., 1994) or directly into the MVN (Gilchrist et al., 1996). In the rabbit vestibular compensation improved with dexamethasone administration in a dose-dependent manner, and was delayed following administration of the glucocorticoid receptor (GR) antagonist RU38486 (Yamanaka et al., 1995b). In the rat RU38486 prevented the increase in intrinsic electrophysiological excitability that takes place in ipsi-lesional MVN neurons after UVL, while dexamethasone administration restored it (Cameron and Dutia, 1999; Johnston et al., 2002). The effects of dexamethasone were observed when it was micro-injected into the ipsi-lesional cerebellar flocculus, indicating that the cerebellum is one site of action for GR activation (Johnston et al., 2002).

### Excessive stress can impair compensation

An optimal level of stress and GR activation seems necessary to facilitate vestibular compensation because additional stress, in the form of restraint applied to a compensating animal after UVD, impedes behavioral recovery and causes de-compensation (the re-appearance of vestibular deafferentation symptoms; Yamamoto et al., 2000). Anecdotally it has been reported that just handling animals after UVD would cause nystagmus to re-appear, perhaps due to stress (Curthoys and Halmagyi, 1995). In addition to the acute de-compensating effects of stress, it should also be noted that neurogenesis and the survival of newly born neurons in the hippocampus is sensitive to stress (Gould et al., 2000; Snyder et al., 2009). Lacour and co-workers have recently provided evidence to suggest that neurogenesis occurs in the cat MVN over a period of time after UVN, and have proposed that this may be involved in vestibular compensation (Tighilet et al., 2007;Dutheil et al., 2009, 2011). Stress may therefore also interact with vestibular compensation by affecting the survival of new neurons in the MVN after vestibular deafferentation, though this remains to be investigated experimentally.

### Stress hormones and neurosteroids affect MVN neuron excitation

Animal studies suggest glucocorticoids facilitate behavioral recovery during vestibular compensation by actions on the medial vestibular nucleus (MVN) and the cerebellum (Seemungal et al., 2001; Paterson et al., 2004; Straka et al., 2005). Prednisone has been shown to excite neurons in the MVN of the cat (Yamanaka et al., 1995a). In addition, neurosteroids can significantly modulate GABAergic and glutamatergic neurotransmission in the MVN. Pregnanolone sulfate and DHEAS reduce GABA receptor efficacy and thus enhance neuronal activity (Yamamoto et al., 1998a,b), while 20-hydroxyecdysone increases GABA activity thus inhibiting neuronal excitability (Okada et al., 1998). Pregnanolone sulfate modulates glutamate receptors and increases MVN neuron activity (Yamamoto et al., 1998b). Tetrahydrodeoxycorticosterone and allopregnanolone also modulate MVN neuronal activity by their actions on GABA and AMPA/kainate receptors (Grassi et al., 2007), while 17 β-estradiol modulates MVN activity by increasing excitatory glutamatergic transmission and reducing GABAergic transmission (Grassi et al., 2009a,b, 2010). These various effects represent a potent and hitherto largely uncharacterized mechanism that regulates the function of the vestibular neural networks. These modulatory effects are likely to be important both in the normal state with basal levels of circulating stress hormones, and the stressed state with elevated levels of stress hormones. In addition, such endocrine modulatory control may mediate the effects of estrogens in menstruating women and potentially explain the increasingly apparent links between the menstrual cycle and the susceptibility to motion sickness, dizziness, and migrainous vertigo (Grassi et al., 2009a).

## Stress and Vestibular Compensation in Man

Dagilas et al. (2005) investigated the stress response evoked by vestibular stimulation in healthy volunteers by measuring serum cortisol levels at the point of maximal nystagmus while undergoing caloric stimulation. Cortisol levels were found to be significantly elevated above resting levels, while the serum concentrations of glutamate, glycine, and GABA were reduced (Dagilas et al., 2005). In a preceding detailed study, Kohl (1985) demonstrated that cross-angular rotatory vestibular stimulation potently stimulated ACTH, noradrenaline, and adrenaline secretion, in a pattern consistent with a vestibular-evoked stress response. Interestingly while this effect was seen in all subjects, individuals with a low susceptibility to motion sickness had higher baseline levels of ACTH as well as a significantly larger response following rotation (Kohl, 1985). The finding that individual stress response profiles may be correlated with the adaptability to stressful motion stimuli (Kohl, 1985) also suggests that in patients with vestibular lesions, the stress response evoked by vestibular deafferentation may be related to their adaptability to the vestibular symptoms, and could influence the subsequent development of vestibular compensation.

### Dimensions of the stress response in humans

While the above focuses on the physiological activation of the HPA axis as an indicator of stress, a more holistic view in recent literature defines stress as “experiences that are challenging emotionally and physiologically” (McEwen, 2007), which normally produce protective and adaptive effects but can also produce deleterious outcomes in certain situations. Stress is regarded as a mechanism of “allostasis,” the process of maintaining homeostatic stability by active means through the production of stress hormones, while “allostatic load or overload” results in wear and tear on the body and brain when this system is dysfunctional (McEwen, 2007). Colloquially this has been termed “good stress and bad stress,” with the former leading to a beneficial outcome while the latter is associated with a failure of homeostatic recovery and the development of abnormal physiological or psychological states. The effects of stress axis activation on vestibular compensation demonstrated in animal studies, where recovery is facilitated by acute stress but impeded by inappropriate stress as discussed above, can be viewed as an example of such an interaction.

### Stress responsiveness and compensation in clinical practice

Vestibular stressors may present either in the form of a single acute episode as in vestibular neuritis, a vascular event or surgical deafferentation, or chronic repeated episodes such as in Benign Paroxysmal Positional Vertigo (BPPV), Meniere’s disease, or migrainous vertigo. If the process of vestibular compensation is inadequate, the underlying dysfunction may persist and eventually present as chronic dizziness (Bronstein et al., 2010). Thus the nature of the stress response in individual patients is likely to vary according to the underlying dysfunction, with an acute episode of vertigo in vestibular neuritis that gradually subsides having a different stress response profile compared with the unpredictable episodes in patients with Meniere’s disease or migrainous vertigo, or the chronic dizziness of the poorly compensated patient.

While animal studies (e.g., in Figure 2) indicate that vestibular imbalance is stressful in itself, the nature of additional stressors that may interact to influence compensation in the human clinical context remain to be determined. As discussed by Joels et al. (2006), a normal stress response within the context of a learning situation focuses attention and improves learning and memory, and this may be a significant role for the vestibular-evoked stress response in facilitating compensation. However for optimal learning to occur the stressor must occur at the same time and act on the same neural circuits (Joels et al., 2006), so that if for example the vestibular stress response competes with an additional stressor such as anxiety, this may prevent vestibular compensation from occurring optimally.

Human imaging and behavioral studies, particularly with reference to spatial memory in patients with loss of vestibular function, suggest the hippocampus may be an important center for vestibular compensation (Brandt et al., 2005; zu Eulenburg et al., 2010). Increased physical activity has been shown in animals to affect brain morphology by promoting neurogenesis in the hippocampus (van Praag et al., 2005) and vestibular exercises and the promotion of physical activity in general may have similar effects in humans. Social support also seems important to alleviate the effects of increased allostatic load by helping humans to cope with acute and chronic stressors (McEwen and Gianaros, 2011). While these are the basic components of any vestibular rehabilitation strategy, further research is needed to clarify the interactions between these interventions and the stress response in relation to compensation.

In addition, of particular relevance is that stress responsiveness is importantly influenced by various developmental and environmental factors including genetic and fetal programming and early life stressful events, as well as varying with age and gender (de Kloet et al., 2005; McEwen, 2007; Raikkonen et al., 2011). Consequently, a significant problem in the study of the interactions between stress and vestibular compensation in humans is the potentially idiosyncratic variation in stress responsiveness between patients, and also in the same patient over time. While animal models offer the potential to isolate and control many of these factors, the application of findings from animal studies to the human clinical context, and the design of effective research paradigms to understand these interactions and their effect on brain plasticity, remains a significant challenge for the foreseeable future. Understanding the factors that optimize a “good” stress response during vestibular rehabilitation to promote compensation, and the extent to which this is altered in anxious patients, deserves further investigation.

### Are steroids of use in early vestibular compensation?

A number of clinical studies have suggested a beneficial role for steroids in the treatment of acute vestibular dysfunction. Ariyasu et al. (1990) studied 20 patients with acute vestibular pathology presenting with vertiginous symptoms within 72 h. Patients were randomized into groups of 10 either receiving a tapering dose of prednisone over 7 days, or placebo. Ninety percent of treated patients reported a decrease in vertigo compared with 30% in the placebo group. Patients in the placebo group that remained symptomatic were switched to the treatment group within 24 h and reported an improvement. All patients with a reduced caloric response at presentation had a normal response when re-tested a month later (Ariyasu et al., 1990).

Kitahara et al. (2003) compared outcomes in 36 vestibular neuritis patients, half of who were prescribed 500 mg methylprednisone in a tapered dose over 1 week. The average time from onset of symptoms to treatment was 2.5 days. No significant difference in improvement in caloric function was detected between the two groups. Subjective outcomes were assessed using a dizziness and unsteadiness questionnaire. Patients treated with steroids were less handicapped due to dizziness when compared to untreated patients (Kitahara et al., 2003).

A systematic study on 141 patients with acute onset vestibular neuritis was conducted by Strupp et al. (2004). Patients were randomized into groups matched for age and sex (placebo – 38, methylprednisolone – 35, valacyclovir – 33, and methylprednisolone with valacyclovir – 35), within hours to days of symptom onset. One-hundred fourteen patients completed the study. The main outcome measure was the change in canal paresis from pre-treatment to 12 months post-treatment. The placebo and valacyclovir-treated groups showed an improvement in canal paresis of 40 and 36% respectively, while there was an improvement of 62% in the methylprednisolone group and 59% in the methylprednisolone with valacyclovir group. The authors concluded that methylprednisolone administration improved the outcome in acute vestibular neuritis, significantly more so than treatment with the anti-viral valacyclovir alone (Strupp et al., 2004).

The effects of prednisolone treatment in vestibular neuritis were investigated by Shupak et al. (2008) in 30 patients from emergency wards and out patient departments. Patients were randomized into a placebo group and a treatment group receiving prednisolone. ENG testing was performed within 3 days of symptoms and only patients with a canal paresis were recruited. Although the caloric response at 12 months showed no difference between the two groups, significantly more patients in the prednisolone-treated group showed complete recovery at 3 and 6 months. Subjective improvement, assessed using the Dizziness Handicap Inventory was not found to be significant (Shupak et al., 2008).

Karlberg and Magnusson (2011) reported a study of 33 consecutive vestibular neuritis patients who were treated within 3 days of symptom onset with 50 mg of prednisolone daily for 5 days, tapered over the next five. Intravenous betamethasone was administered on day 1 and 2 if nausea prevented the use of oral medication. Caloric function at 1 year and length of hospital stay were compared, with historic controls receiving no treatment. Seventy percent of the treated patients recovered caloric function at 1 year compared with 34% of controls, and hospital stay was also shorter in the group treated with steroids (Karlberg and Magnusson, 2011).

Two recent meta-analyses have reviewed the role of steroids in the treatment of acute vestibular dysfunction (Goudakos et al., 2010; Fishman et al., 2011). Although both studies reviewed predominantly the same papers, there were differences in outcomes. While Goudakos et al. use the term “vestibular neuritis,” Fishman et al. prefer to use “idiopathic vestibular dysfunction,” suggesting that all cases may not be virally induced and therefore inflammatory. This is important when considering the mechanism of action of steroids, as in this instance their actions can include both anti-inflammatory effects as well as effects on central pathways involved in vestibular compensation. Each meta-analysis included four placebo-controlled studies, of which three were common (Ariyasu et al., 1990; Strupp et al., 2004; Shupak et al., 2008). The outcome measure compared between studies was the complete recovery or improvement in canal paresis at 12 months. While Goudakos et al. (2010) found an improvement using a fixed effect model, Fishman et al. (2011) found to the contrary using a random-effects model. Attention was drawn to the relatively small sample sizes, and differences in the clinical, methodological, and statistical details between the studies. Nonetheless from the current literature there is sufficient evidence to warrant further carefully considered trials.

### Chronic stress and the poorly compensated patient

Horii et al. (2007) reported elevated cortisol levels in dizzy patients with neuro-otological diagnoses compared with those in a group of patients with idiopathic dizziness, whereas plasma AVP levels were found to be elevated in both groups (Horii et al., 2007). Takeda et al. (1995) also reported high AVP levels in patients during an acute attack of Meniere’s disease (Takeda et al., 1995). van Cruijsen et al. (2005) reported significantly elevated serum and salivary cortisol levels in Meniere’s patients compared with healthy controls (van Cruijsen et al., 2005). Horner and Cazals (2005) assessed cortisol and ACTH in patients with Meniere’s disease and vestibular schwannoma and compared them with levels in patients with hemifacial spasm (as controls), and reported a strong positive correlation between ACTH and cortisol in patients with vestibular schwannoma and Meniere’s disease, but no such correlation in patients with hemifacial spasm. This suggests a chronic stress response in patients with persistent vestibular dysfunction (Horner and Cazals, 2005). In a prospective longitudinal study of the relationship between stress and the symptoms of Meniere’s disease over a period averaging 7 months, Andersson et al. (1997) found that dizziness was the symptom most associated with stress. However there was no evidence that stress on preceding days was responsible for the symptoms, suggesting that chronic stress could be the result of symptoms rather than the cause (Andersson et al., 1997).

In patients with chronic bilateral vestibular loss, testing with virtual navigation tasks without self motion revealed significantly impaired spatial memory that was accompanied by a reduction in hippocampal volume on MRI imaging (Brandt et al., 2005). It is well established that patients with chronic stress undergo hippocampal remodeling and display hippocampal atrophy with deficits in spatial memory (McEwen, 2001; Kim and Diamond, 2002). However, vestibular inputs are also known to be very important in the dynamic spatial tuning of hippocampal place cells (Stackman et al., 2002; Russell et al., 2006). Thus the loss of vestibular input, as well as chronic stress, may synergize in causing the deleterious effects on hippocampal function and total volume in patients.

### Stress, anxiety, and vestibular compensation

Eagger et al. (1992) reported on 54 patients with a history of disequilibrium, objective evidence of a peripheral vestibular lesion and current neuro-otological symptoms. Patients underwent a psychiatric assessment and completed The Social Stress and Support Interview Schedule (Jenkins et al., 1981) which explores the degree of stress and support experienced by the patient in various facets of everyday life. Patients with vestibular symptoms and psychiatric morbidity were found to present with significantly increased anxiety scores and experienced a greater degree of social stress (Eagger et al., 1992).

Arousal with aversion and a lack of control are behavioral hallmarks of the stress response (Kim and Diamond, 2002). In a longitudinal study, Yardley et al. (1994) found that autonomic symptoms or arousal and somatic anxiety were the best predictors of reported change in levels of vertiginous symptoms. It was postulated that autonomic symptoms initially associated with a vertiginous attack cause anxiety and further arousal, which worsens the vertigo in a vicious cycle (Yardley et al., 1994).

In a cross-sectional study Eckhardt-Henn et al. (2008) found that patients with Meniere’s and vestibular migraine had a significantly higher risk of psychiatric disorders such as anxiety and depression when compared with vestibular neuritis, BPPV, and normal groups. The unpredictability and lack of control of the attacks in Meniere’s and vestibular migraine, as opposed to the symptoms in BPPV and vestibular neuritis, may be the likely reason for the increase in psychiatric morbidity (Eckhardt-Henn et al., 2008). Vreeburg et al. (2010) demonstrated in a cross-sectional study using a large cohort that anxiety, in particular panic disorder, was associated with increased levels of cortisol (Vreeburg et al., 2010), which reinforces the positive-feedback cycle suggested above. The anatomical substrates that may mediate this relationship between balance, anxiety, and stress have been postulated by Balaban and Thayer (2001) and Balaban (2002). In particular the nucleus tractus solitarii have extensive relationships with the vestibular nuclei both via direct projections and indirectly through the parabrachial nucleus, which provides a major input into the limbic system including the extended central amygdaloid nucleus, the infralimbic cortex, and the hypothalamus (Balaban and Thayer, 2001; Balaban, 2002).

However, not all patients perceive or handle stress in the same way. Patients’ ability to habituate or cope with repeated stress may be factors that determine outcomes. Tschan et al. (2011) assessed resiliency (the ability to cope successfully with life change or misfortune) and coherency (subjective perception that enables people to manage stressful stimuli) as factors that predict the development of somatoform vestibular dizziness in patients 1 year after admission. Their cohort included patients with BPPV, Meniere’s, vestibular neuritis, and vestibular migraine. Patients who developed somatoform dizziness showed significantly more lifetime mental disorders, mental co-morbidity, and stressful life events compared to patients who recovered normally. Significantly, patients who recovered normally had higher scores on the resiliency and coherency questionnaires, suggesting that they displayed better coping mechanisms with regard to stress. Thus the longer-term outcomes of vestibular dysfunction or damage may be rather idiosyncratic, likely to be influenced not only by the nature of the associated stress response in each patient but also by the coping mechanisms that they are able to bring into play (Tschan et al., 2011).

## Conclusion

The causal relationships between balance dysfunction, anxiety, and stress need to be explored experimentally especially within the context of vestibular compensation. An acute stress response is known to be essential for learning and memory formation (Joels et al., 2006) and may be an important component of vestibular rehabilitation and compensation. However repeated exposure to stressful stimuli or chronic stress can lead to an inhibition of brain plasticity and lasting detrimental changes in the hippocampus, amygdala, and prefrontal cortex (de Kloet et al., 2005; McEwen, 2007; Roozendaal et al., 2009). A range of factors influence the responsiveness of an individual to stress, including age, sex and genetic factors, fetal programming, and early life stressful experiences, so that in the human clinical context the vestibular-evoked stress response may be highly personalized and idiosyncratic. An aberrant acute stress response elicited by vestibular symptoms may, in susceptible individuals, affect the process of central compensation, possibly leading to lasting deficits. It also seems plausible from clinical and animal studies that augmenting the acute response with exogenous steroids may be beneficial. Further studies of the interactions between stress and vestibular system plasticity in animals and man are necessary to fully understand their importance in functional recovery after vestibular lesions.

## Conflict of Interest Statement

The authors declare that the research was conducted in the absence of any commercial or financial relationships that could be construed as a potential conflict of interest.
